# Effects of Ontogeny on δ^13^C of Plant- and Soil-Respired CO_2_ and on Respiratory Carbon Fractionation in C_3_ Herbaceous Species

**DOI:** 10.1371/journal.pone.0151583

**Published:** 2016-03-24

**Authors:** Yann Salmon, Nina Buchmann, Romain L. Barnard

**Affiliations:** Institute of Agricultural Sciences, ETH Zurich, Zurich, Switzerland; University of Copenhagen, DENMARK

## Abstract

Knowledge gaps regarding potential ontogeny and plant species identity effects on carbon isotope fractionation might lead to misinterpretations of carbon isotope composition (δ^13^C) of respired CO_2_, a widely-used integrator of environmental conditions. In monospecific mesocosms grown under controlled conditions, the δ^13^C of C pools and fluxes and leaf ecophysiological parameters of seven herbaceous species belonging to three functional groups (crops, forage grasses and legumes) were investigated at three ontogenetic stages of their vegetative cycle (young foliage, maximum growth rate, early senescence). Ontogeny-related changes in δ^13^C of leaf- and soil-respired CO_2_ and ^13^C/^12^C fractionation in respiration (Δ_R_) were species-dependent and up to 7‰, a magnitude similar to that commonly measured in response to environmental factors. At plant and soil levels, changes in δ^13^C of respired CO_2_ and Δ_R_ with ontogeny were related to changes in plant physiological status, likely through ontogeny-driven changes in the C sink to source strength ratio in the aboveground plant compartment. Our data further showed that lower Δ_R_ values (i.e. respired CO_2_ relatively less depleted in ^13^C) were observed with decreasing net assimilation. Our findings highlight the importance of accounting for ontogenetic stage and plant community composition in ecological studies using stable carbon isotopes.

## Introduction

The carbon isotopic composition (δ^13^C) of plant- and soil-respired CO_2_ is often used to infer plant physiological responses (e.g., review by [1]) and compare the responses of plants and ecosystems to changes in environmental conditions (e.g., [2]). However, our understanding of the biological mechanisms generating the C isotopic signature of plant and soil respiration fluxes is still incomplete, and the interpretation of the ^13^C signature of respired CO_2_ can be challenging since this isotopic signal is affected by both environmental and biological factors. In particular, the effects of ontogeny and plant species identity on respiratory signatures in the plant-soil continuum remain poorly understood. Indeed, these biological factors are often difficult to separate from environmental factors, since 1) both ontogeny and environmental conditions change over the growing season, and 2) species-specific effects can be confounded with environmental differences between studied systems.

Differentiating biological from environmental effects on the isotopic signature of plant-soil continuum components requires controlling either one or the other. To date, this has been tackled in only a few studies under controlled environmental conditions. However, effects of ontogeny (e.g., [3]) or species identity (e.g., [4]) have been suggested to be major drivers of change in δ^13^C in real plant communities. For example, large ontogeny-related changes in the carbon isotopic signature of plant tissues (up to 10‰) have been previously observed for herbaceous species [5]. Furthermore, plant communities are rarely monospecific, and different species are not expected to have synchronous ontogeny. Therefore, it is crucial to understand the interaction between ontogeny and species identity when studying changes in isotopic signature of plant-soil continuum.

Furthermore, the δ^13^C of plant- and soil-respired CO_2_ is the average of the δ^13^C of their different components (i.e., leaves, stems, roots, soils, soil microorganisms), all of which are driven by respiratory fractionation (Δ_R_, see appendix for list of abbreviations). Thus, δ^13^C of respired CO_2_ is a powerful tool to understand biochemical pathways of plant organs [3, 6–11]. Ontogeny and species identity have been predicted to also affect respiratory processes that are involved in controlling the δ^13^C of CO_2_ respired by the different components of the plant-soil continuum (leaf, stem, soil, root, soil microorganisms [3, 4, 12]) as well as the associated respiratory fractionation [12]. As plants grow older, a larger contribution of maintenance vs. growth respiration to the respiration fluxes of their organs may result in changes of the relative importance of the underlying metabolic pathways, leading to changes in Δ_R_ [13]. Recent studies (e.g., [14]) further suggest that the ratio between C acquisition (assimilation; i.e. C sources) and C loss (respiration; i.e. C sinks) plays a major role in controlling processes that affect Δ_R_.

The molecular mechanisms underlying respiratory fractionation have been widely studied in the last decade (e.g., [12, 15]). The mechanisms controlling Δ_R_ that may be affected as plants age include 1) temporal changes in photosynthetic discrimination and use of different substrates, which can affect the intrinsic ^13^C signature of the respiratory substrate ([13] and references therein); 2) processes taking place after C assimilation, also affecting the isotopic signature of respiratory substrates (e.g., post-carboxylation discrimination, daytime respiration and photorespiration, see [16] and references therein); and 3) changes in metabolic pathways and thus fractionation by respiratory enzymes. Furthermore, the relative contribution of these different processes likely differs between leaves (C source organs) and C sink organs as well as among C sink organs. In addition, in young leaves, the change from heterotrophy to autotrophy, followed–once photosynthesis has started–by changes in photosynthetic discrimination due to the modification of both internal (e.g., mesophyll) and external (e.g., stomatal) conductances as the leaf matures, will modify the isotopic signature of the C source for respiration. However, despite increasing evidence that ontogeny may strongly influence the isotopic signature of plant tissues and respired CO_2_ (e.g., [3, 5, 17–19]), few data are actually available to assess the effect of biological controls over Δ_R_.

The present study focused on the effect of ontogeny and species identity on the isotopic signature of respired CO_2_ and the associated Δ_R._ Furthermore, we tested whether changes in δ^13^C of leaf- and soil-respired CO_2_ and Δ_R_ could be related to leaf gas exchange and soil CO_2_ efflux, used as proxies for C source and sink activities. Seven temperate C_3_ grassland and cropland plant species were grown in monoculture under controlled conditions over a whole life cycle. The δ^13^C of leaf- and soil-respired CO_2_ as well as the δ^13^C of plant organs were used to determine the apparent respiratory fractionation during leaf and soil respiration, and were related to changes in leaf gas exchange, soil CO_2_ emissions, and plant biomass. Our objectives were: 1) to assess the effects of ontogeny on the δ^13^C of CO_2_ respired by plants and soil, 2) to estimate the associated Δ_R_ and whether these ontogeny effects may be species-specific; and 3) to determine the relationship between δ^13^C of respired CO_2_, Δ_R_ and leaf gas exchange and soil CO_2_ emissions over a range of growth stages.

## Material and Methods

### Experimental setup

This experiment included seven C_3_ herbaceous species covering three different functional groups, based on *a priori* plant biological characteristics related to C and nitrogen allocation strategies: *Arrhenatherum elatius* L., *Dactylis glomerata* L., *Lolium perenne* L. (forage grasses), *Hordeum vulgare* L., *Triticum aestivum* L. (crop grasses bred for grain production, expected to favour aboveground productivity and allocation to seeds), *Medicago sativa* L., *Trifolium pratense* L. (legumes, less prone to nitrogen limitation, but likely allocating significant amounts of C to their microbial partner in the symbiosis). Square pots (18x18x17 cm height) were filled with sieved (1 cm mesh) clay loam soil (30% clay, 41.8% silt, 28.1% sand; pH 6.8, organic matter content 28.5%). One week after seed germination on a thin layer of soil, the plantlets were transferred to the pots, following an even pattern (16 individuals in monoculture per pot, i.e., 658 plants m^-2^) on three quarters of the pot area. Despite the high plant density, we did not find any indication that root growth was impaired at any stage of the experiment. A PVC collar (7 cm diameter, 5 cm high) for soil CO_2_ efflux measurements was inserted 2.5 cm deep in the remaining quarter of the pot surface.

Plants were grown under similar and optimal conditions in six growth chambers (PGV36, Conviron, Winnipeg, Canada) set to a 14 hour light period (photosynthetically active radiation of ca. 400 μmol m^-2^ s^-1^) at 20°C and an 8 hour dark period at 15°C, with two gradual 1h transitions between light and dark periods. To avoid position effects, pots were randomly distributed within each chamber, and their position within the chambers was changed weekly. Additionally, enough space was left between the pots to avoid shading from taller species over smaller ones. The spacing was increased at each stage and facilitated by the removal of one third of the pots at each stage for measurements and sampling (see below). The chambers were not airtight: outside air was continuously pumped in from above the roof of the building to the room with the growth chambers–which acted as a large buffer from which the growth chamber pumped their air from–, resulting in chamber CO_2_ concentration that was monitored to be at approximately 400 μmol mol^-1^ (±10%), close to equilibrium with atmospheric CO_2_ concentration. Air humidity was maintained between 60 and 70%.

Three vegetative ontogenetic stages were defined for each species prior to the experiment: 1) young foliage, where plants had autotrophic leaves and were just large enough (10 cm high) to be used for our measurements (1 to 4 weeks after germination, depending on the species; termed “young”), 2) maximum growth rate, based on weekly measurements of plant height increase (5 to 8 weeks; termed “mature”), and 3) beginning of senescence (8 to 10 weeks; termed “old”), dominated by maintenance respiration. All measurements were performed on non-senescent stems and leaves. These three growth stages were expected to be associated with different contributions of growth and maintenance respiration to total respiration [8], ranging from mostly growth respiration at the young stage due to the limited maintenance requirements of the small existing biomass, to both growth and maintenance respiration contributing to total respiration at the mature stage, and finally to mostly maintenance respiration at the old stage, during which biomass is no longer produced. Due to growth conditions of constant photoperiod and temperature, flowering and seed production were not initiated during this experiment.

### Leaf-gas exchange measurements

The following ecophysiological variables were measured on fully expanded leaves of three plant replicates (i.e., growing in three different climate chambers) per ontogenetic stage and species, between the 6^th^ and the 8^th^ hour of the light period: transpiration rate (E), stomatal conductance of leaves to H_2_O in the light (g_s_) as well as net CO_2_ assimilation rate (A). Additionally, leaf respiration rate (R_l_) was measured at the end of the dark period (between 5^th^ and 7^th^ dark hour) to avoid light-enhanced dark-respiration [20]. Five measurements were averaged per replicate. Measurements were carried out under standardised conditions with a portable photosynthesis system (Li-6400, Li-Cor Inc., Lincoln, NE, USA). A dewpoint generator (Li-610, Li-Cor Inc.) and a CO_2_ source ensured constant relative humidity (60%) and CO_2_ concentration (400 μmol mol^-1^) in the incoming flow of the Li-6400 leaf chamber. Temperature and light conditions were set to growth chamber conditions: 20°C, 400 μmol m^-2^ s^-1^ (light source: 6400-02B, Li-Cor Inc.). Leaf gas exchange was measured using a leaf chamber covering 6 cm^2^ of leaf area. When the leaf was too small to cover the whole chamber, leaf area was determined after leaf gas exchange, using a portable area meter (Li-3000C, Li-Cor Inc.), and the gas exchange values were recomputed for the correct leaf area. Total leaf area (cut 1 cm above the ground) was measured (portable area meter LI-3000C) before drying (48h at 60°C) and weighing.

The soil CO_2_ efflux rate was measured on three replicates (i.e., growing in three different climate chambers) per ontogenetic stage and plant species. A custom-made PVC chamber (27 cm high, 14.5 cm diameter) was fitted on the collar installed in the pots, and connected to a CO_2_/H_2_O gas analyser (Li-840, Li-Cor Inc.). The soil CO_2_ efflux rate was calculated over 1 minute of linear CO_2_ concentration increase in the chamber.

### Sample collection

Leaves, roots, soil, and phloem sap organic matter were collected (n = 6 replicates per ontogenetic stage and species combination, i.e. one per growth chamber) between the 3^rd^ and 5^th^ hour of the dark period. Note that these measurements preceded R_l_ measurements by 2 hours, during which some C export to phloem may have occurred. However, the potential offset between measured respiration rate and isotopic signature of phloem sap organic matter should be small under our experimental conditions [7]. The individuals sampled for organic matter were different from those used for leaf gas exchange measurements to avoid any confounding effects of the δ^13^C value of the CO_2_ source used during leaf gas exchange measurements. Aboveground biomass was cut 1 cm above the root crown. A first soil core (1.5 cm diameter core over the whole pot depth) was taken for bulk soil δ^13^C measurements, after manually removing roots. A second soil core (5 cm diameter core over the whole pot depth) was taken for microbial biomass δ^13^C measurements (see below). Roots were separated from the soil remaining in the pot by wet sieving. Leaf, root and soil samples for isotope composition analysis were dried (48h at 60°C) and finely ground (see below). Gravimetric soil water content was calculated after drying approximately 10 g soil at 105°C. Bulk phloem sap organic matter was collected by using an exudation method [21] adapted for herbaceous plants [5]. Briefly, for each species and ontogenetic stage replicate, one to three stems (or pseudostems) were cut at 1 cm height, rinsed with ultrapure water and carefully dabbed. Then, stems were inserted in a tube filled with 2 mL of 0.15M polyphosphate buffer at pH 7.5, sealed with parafilm® and placed in the dark (100% humidity, 4°C). After five hours, 1.5 mL of solution were collected, lyophilized, and used for C isotope composition analysis (see below).

For collection of soil CO_2_, a soil chamber (Li-6400-09, Li-Cor Inc.) was adapted to fit the collars installed in all pots. For collection of leaf respired CO_2_, a custom-made PVC chamber (15x7x4 cm) was used. The chamber was connected to the closed path infrared gas analyser of the Li-6400 portable photosynthesis to monitor chamber [CO_2_]. Both chambers were equipped with a septum to allow sampling CO_2_ respired by soil and leaves for isotope analysis.

C isotopic signatures of CO_2_ respired by soil and leaves (individual leaves, not total aboveground biomass) were calculated using a Keeling plot approach. Briefly, this is a two end-member mixing model between the CO_2_ emitted by a source and the background atmospheric CO_2_, which allows to determine the isotopic signature of the source [22]. If more than two components mix, it is however possible to integrate several of them as one, provided they are well mixed and their relative contribution to the overall flux remains constant over the sampling period [23]. For example, belowground autotrophic respiration and heterotrophic respiration can be considered as an overall soil respiration component when using soil chambers (see below) The mixing model was based on five samples of chamber air, collected at regular intervals over a [CO_2_] increase of at least 100 μmol mol^-1^, and injected in vials (Exetainer®, Labco Ltd, High Wycombe, UK) that had been previously evacuated (<4.10^3^ Pa) and filled with N_2_. Sample volume and vial volume for each measured compartment were chosen to represent less than 1% of the chamber volume and be within a practical [CO_2_] range for isotope composition measurements (soil: 5 mL sample, 2.7 mL vial; leaf: 3 mL sample, 2 mL vial). All samples were collected between the 3^rd^ and 5^th^ hour of the dark period, thus effectively avoiding light-enhanced dark-respiration [20]. Vials were stored in a CO_2_-free environment and measured for δ^13^C within 48h.

To allow comparison between samples taken at different times, all δ^13^C measurements were corrected for changes in background δ^13^C, weighed by the duration of each stage. Background δ^13^C was measured in a companion study [5] and the values are provided in S1 Table (δ^13^C_air_).

Soil microbial biomass was extracted by fumigation-extraction [24] after soil sieving (2 mm mesh [24, 25]). For each soil sample, one 10 g subsample was fumigated 24 h with chloroform vapour before extracting microbial biomass, while a second 10 g subsample was extracted without prior fumigation. Microbial C was extracted by vigorous shaking for 30 minutes in 30 mM K_2_SO_4_ solution, then the extracts were filtered, frozen (-18°C) and lyophilised before isotope composition and C concentration analyses.

### Isotope ratio mass spectrometry measurements

The measurement of δ^13^C values and C concentrations of plant biomass, soil and phloem sap organic matter was performed using a Flash EA 1112 Series elemental analyzer (Thermo Italy, former CE Instruments, Rhodano, Italy) coupled to a Finnigan MAT Delta^plus^XP isotope ratio mass spectrometer (Finnigan MAT, Bremen, Germany) via a 6-port valve [26] and a ConFlo III [27]. The positioning of samples, blanks and laboratory standards in a measurement series followed the principle described by Werner and Brand [28]. Post-run off-line calculations like blank-, offset- and possibly drift-corrections for assigning the final δ^13^C values on the V-PDB scale were performed according to Werner and Brand [28]. Calibration of laboratory standards (acetanilide, caffeine, tyrosine) was periodically performed by comparing them to the corresponding international reference materials (NBS 22, USGS24) provided by the IAEA (Vienna, Austria). The long-term precision (~1.5 years) of our quality control standard (caffeine) was 0.09 ‰ or better for δ^13^C.

The δ^13^C values of CO_2_ derived from gaseous samples were measured with a modified Gasbench II periphery (Finnigan MAT, Bremen, D) coupled to an isotope ratio mass spectrometer (Delta^plus^XP; Finnigan MAT). The modification of the Gasbench comprises the replacement of the GC-type split by a ConFloIII-like split and the addition of a home-built cold trap (filled with Ni-wire) instead of the loop of the 8-port valve inside the Gasbench (modification as described by Zeeman et al. [29]).

Post-run off-line calculation like offset- and drift correction for assigning the final δ^13^C values on the V-PDB scale were done as described above. The δ^13^C values of the laboratory CO_2_-in-air standards were determined at the Max-Plack-Institut für Biogeochemie (Jena, Germany) as described by Werner et al. [28]. The measurement of the aliquots of the laboratory standards is routinely better than 0.15 ‰.

C isotopic composition is expressed as the relative difference of the isotope abundance ratio of a sample relative to that of the VPDB international standard. This difference is expressed in per mil and defined as:

δ13C[‰]=[(C13/C12)sample(C13/C12)VPDB−1] (1)

δ^13^C value of atmospheric CO_2_ over each ontogenetic stage was calculated using phytometer leaves (see [5]).

For clarity, we use the following notation depending on whether the product is 1) the bulk δ^13^C value of a given plant part (δ^13^C_p_: δ^13^C_leaf_, δ^13^C_root_, δ^13^C_phloem_) or of soil microbial biomass (δ^13^C_mic_) or 2) the δ^13^C value of respired CO_2_ by either leaf or soil CO_2_ efflux (δ^13^C_CO2_: δ^13^C_CO2-leaf_, δ^13^C_CO2-soil_).

δ^13^C_mic_ was calculated using the following mass balance equation:

$$\delta^{13}C_{mic}=\frac{\delta^{13}C_{F}\times C_{F}-\delta^{13}C_{NF}\times C_{NF}}{C_{F}-C_{NF}}$$ (2)

where δ^13^C_F_ and δ^13^C_NF_ are the δ^13^C values measured in fumigated and non-fumigated subsamples, respectively, and C_F_ and C_NF_ are C concentrations in fumigated and non-fumigated subsamples, respectively.

### Carbon isotope discrimination

C isotope discrimination during a biochemical reaction (i.e., from a source to a product) can be calculated according to Farquhar *et al*. [30]:

$$\Delta =\frac{\delta_{source}-\delta_{product}}{1+\delta_{product}}$$ (3)

where Δ is the discrimination between δ_source_ and δ_product_, the δ^13^C values of the source and the product of the reaction, respectively. Unlike δ values, discrimination is independent of the isotopic composition of the source.

Respiratory fractionation (Δ_Rsubstrate-product_) is estimated as discrimination between the δ^13^C value of CO_2_ respired (either leaf or soil) and its putative substrate:

$$\Delta_{\;Rsubstrate-product}=\frac{\delta_{S}-\delta_{CO2-X}}{1+\delta_{CO2-X}}$$ (4)

where δ_s_ is the δ^13^C value of the putative substrate (i.e. phloem sap organic matter in the main part of the study) and δ_CO2-X_ is the δ^13^C value of the CO_2_ respired by X. We use the explicit term Δ_Rsubstrate-product_, based on “Δ_R_” used in previous studies [31].

Since phloem is the main source of C for non-autotrophic tissues and its isotope signature a good proxy for total canopy C discrimination [32], we chose to present and discuss respiratory C isotope fractionation between leaf-respired CO_2_ and phloem sap organic matter (Δ_Rphloem-leaf_). Nevertheless, respiratory fractionation was also calculated based on other C pools (i.e., leaf biomass) that may represent proxies for the actual leaf respiration substrate. Since CO_2_ efflux from the soil has multiple sources and quantifying their relative contributions was beyond our objectives, we chose to calculate belowground respiratory C isotope fractionation between soil CO_2_ efflux and bulk phloem sap organic matter (Δ_Rphloem-soil_). However, as we did for leaf respiration, we also calculated soil respiratory fractionation based on other C pools that may represent proxies to soil respiration (i.e., leaf biomass and root biomass).

C isotope discrimination during photosynthesis is predicted from non-isotopic gas exchange measurements based on the widely accepted simplified model developed by Farquhar *et al*. [30], that assumes infinite internal conductance and neglects the effect of (photo)respiration, and further referred to as predicted photosynthetic C isotope discrimination (Δ_i_):

$$\Delta_{i}=a+(b-a)\frac{C_{i}}{C_{a}}$$ (5)

where a is the fractionation occurring during CO_2_ diffusion in air through the stomatal pore (a = 4.4‰, [33]), b is the net fractionation caused by carboxylation, c_a_ and c_i_ are ambient and substomatal partial pressures of CO_2_, respectively. For higher C_3_ plants, b mostly results from the fixation of CO_2_ by RuBisCO, the carboxylation enzyme, estimated at 29‰ in spinach [34]. Additional fractionation effects resulting from PEP-carboxylase fixation, CO_2_ transfer through the leaf boundary layer, dissolution of CO_2,_ liquid-phase diffusion (in the mesophyll cells), photorespiration and dark respiration during photosynthesis are gathered under the term *d* [35], which–when neglected–typically leads to the use of b = 27‰ ([16] see also [36]), as commonly used in ecological studies [37, 38] and also used in the present study. Note that *d* was discussed in detail in our companion study [5].

### Statistical analysis

Data were analysed using R 3.0.2 [39]. The effects of ontogeny and species identity were tested by analysis of variance, nesting species identity within the functional group, and including growth chambers as blocks to remove any chamber-related effects from the analyses. Differences of means among groups were tested with a Tukey honest significant difference test (HSD). Linear regression models were used to fit regression coefficients.

## Results

### Leaf gas-exchange

Species, functional group and ontogeny had significant effects on all leaf gas exchange variables (S2 Table). Furthermore, the effect of ontogeny differed among species (significant ontogeny × species interaction): in particular, leaf respiration (R_l_) varied by an order of magnitude among different plant species and ontogenetic stages growing under controlled conditions (Table 1). Leaf respiration rates were the highest in mature *Medicago* foliage (1.68 μmol m^-2^ s^-1^), and the lowest in old *Hordeum* leaves (0.16 μmol m^-2^ s^-1^). A and g_s_ data were previously published in Salmon *et al*. [5]. Assimilation was highest in mature *Triticum* leaves (14.45 μmol m^-2^ s^-1^) and lowest in old *Hordeum* leaves (5.63 μmol m^-2^ s^-1^). Stomatal conductance values ranged from 43.8 mmol H_2_O m^-2^ s^-1^ (old *Hordeum*) to 708.1 mmol H_2_O m^-2^ s^-1^ (young *Triticum*). Resulting Δ_i_ ranged from 14.8‰ to 26.3‰, with an overall average of 23.4±0.6‰ [5]. Aboveground biomass and leaf area at the whole pot level both increased with age for all species (p<0.001), except for *Trifolium* that was more advanced in senescence and had already lost leaves between mature and old stages (Table 1). Soil CO_2_ efflux rate (R_s_) also varied among species and ontogenetic stages (0.6 to 44.3 μmol m^-2^ s^-1^, p<0.001). *Arrhenatherum*, *Dactylis* and *Trifolium* exhibited high soil CO_2_ efflux rates at mature and old stages, like *Medicago* and *Lolium* at the old stage.

**Table 1:** Leaf respiration rate (R_l_), net assimilation rate (A), leaf stomatal conductance to H_2_O (g_s_), instantaneous predicted photosynthetic discrimination (Δ_i_), aboveground biomass at the whole pot level (B), leaf area at the whole pot level (LA) and soil respiration rate (R_s_) for different plant species at three ontogenetic stages (young foliage, maximum growth rate and beginning of senescence, indicated by young, mature and old, respectively). A, g_s_ and Δ_i_ (recalculated with b = 27‰) values are from Salmon et al. (2011). Values are mean±1SE (n = 3).

Ontogenetic	Species	R_l_	A	g_s_	Δ_i_	B	LA	R_s_
stage		[μmol m^-2^ s^-1^]	[μmol m^-2^ s^-1^]	[mmol H_2_O m^-2^ s^-1^]	[‰]	[g]	[cm^2^]	[μmol m^-2^ s^-1^]
Young								
	*Arrhenatherum*	0.68±0.01	8.8±0.9	334.4±57.1	24.0±0.8	0.15±0.03	36.8±8.6	2.3±1.3
	*Dactylis*	0.41±0.03	12.01±0	355.9±4.7	23.2±0.1	0.55±0.06	107.6±9.6	4.1±0.5
	*Hordeum*	0.93±0.07	13.97±1.7	512.2±11.5	25.5±1.2	0.48±0.06	202.9±19.2	2±0.4
	*Lolium*	1.04±0.01	10.04±1.9	354.4±135.6	25.5±1.8	0.28±0.03	71±4.2	4±0.8
	*Medicago*	0.8±0.01	10.11±1.6	329.9±31.7	25.2±2.4	0.27±0.01	73.6±4	2.8±1.3
	*Trifolium*	1.38±0.01	11.46±0	180.7±0.2	21.9±0.0	0.26±0.03	98.5±7.1	3±1.1
	*Triticum*	0.85±0.01	13.79±1.4	708.1±24.5	26.3±0.7	0.38±0.03	164.4±10.6	1.2±0.4
Mature								
	*Arrhenatherum*	0.84±0.13	10.44±2.1	171.7±51.4	20.1±1.0	7.15±0.83	2975.6±191.6	25.1±3.7
	*Dactylis*	0.49±0.06	6.02±1.2	75.8±15.5	20.5±0.6	6.26±0.39	1174.7±184.7	19.4±4.3
	*Hordeum*	0.78±0.04	13.12±0.5	515.4±60.3	25.7±0.3	3.95±0.39	1345.6±130.8	1.4±0.2
	*Lolium*	0.61±0.06	7.72±0.5	163.3±19.4	23.5±0.3	5.54±0.18	1253±69.4	0.8±0.2
	*Medicago*	1.68±0.25	11.33±0.4	249±26.4	23.5±0.5	1.01±0.03	490.9±25.6	0.6±0.1
	*Trifolium*	1.09±0.18	11.17±0.8	213.1±12.9	23.0±0.1	4.25±0.58	1306.6±224.6	21.6±0.9
	*Triticum*	0.71±0.05	14.45±0.5	625.7±50.4	25.9±0.4	2.64±0.34	918±79.7	1.1±0.1
Old								
	*Arrhenatherum*	1.28±0.33	7.33±1.2	182.7±31.4	22.6±0.2	9.62±1.69	4178.8±1044.4	26.1±10.3
	*Dactylis*	1.33±0.17	8.83±0.7	176.8±13.9	23.2±0.1	12.21±1.53	3375.1±412.2	26.7±7.8
	*Hordeum*	0.16±0.03	5.63±1.2	43.8±14.2	14.8±1.2	8.61±0.73	1767.6±473.9	1.1±0.2
	*Lolium*	0.46±0.1	7.04±0.3	97.1±15.6	20.8±1.0	9.52±0.78	2066.6±228.2	20.3±2.1
	*Medicago*	0.58±0.08	11.19±0.6	150.1±8	20.7±0.3	5.8±0.58	1641.2±70	41.1±11.8
	*Trifolium*	0.98±0.27	14.35±1.17	464.9±62.2	25.0±0.4	3.7±0.5	934.4±161.8	44.3±29.8
	*Triticum*	0.27±0.07	11.37±1.1	227.6±100	21.3±2.2	10.4±0.84	2418.6±447.2	2±0.1

### Isotopic signature of plant tissues and leaf-respired CO_2_

δ^13^C_leaf_, δ^13^C_phloem_ and δ^13^C_root_ values were previously published [5]. The isotopic signature of leaf biomass (δ^13^C_leaf_) was on average -31.1±0.3‰ (mean±SE, S1 Table), significantly lower than phloem sap organic matter (δ^13^C_phloem_, -30.3±0.3‰, 0.001<p≤0.01) and root biomass (δ^13^C_root_, -29.4±0.4‰, p<0.001). Ontogeny and species significantly affected δ^13^C_leaf_, δ^13^C_phloem_ and δ^13^C_root_ (p<0.001 in all three cases). The effect of ontogeny differed among species (significant ontogeny × species interaction) for all δ^13^C_p_ (p<0.01), except for δ^13^C_phloem_.

**Fig 1:**
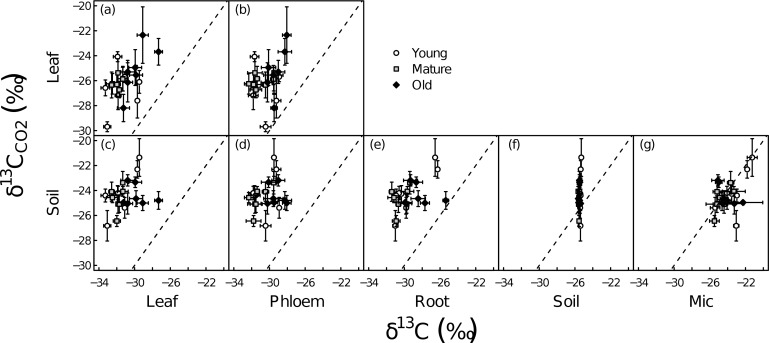
δ^13^C of leaf-respired CO_2_ compared to δ^13^C of aboveground C pools, i.e., leaf biomass (a) and phloem sap organic matter (b), and δ^13^C of soil-respired CO_2_ compared to above and belowground C pools, i.e., leaf biomass (c), phloem sap organic matter (d), root biomass (e), bulk soil (f) and microbial biomass (g) at three ontogenetic stages (young foliage, “young”, white circles; maximum growth rate, “mature”, grey squares; beginning of senescence, “old”, black diamonds). Each point represents the average value (n = 6) for a given plant species at a given ontogenetic stage. Errors bars indicate ±1SE, the dashed line represents y = x.

**Fig 2:**
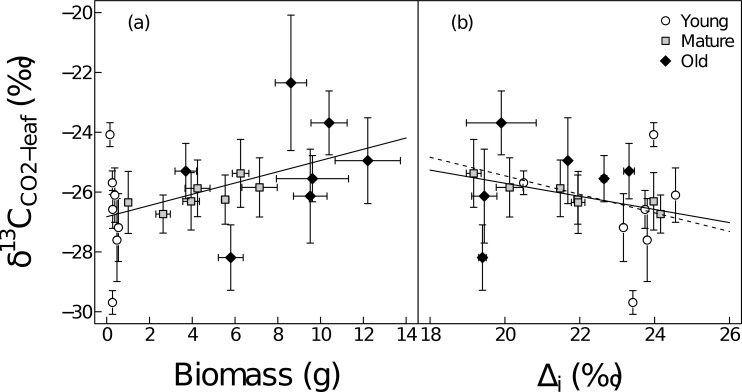
Response of δ^13^C values of leaf-respired CO_2_ (δ^13^C_CO2-leaf_) to leaf biomass (a) and to Δ_i_ (b) at three ontogenetic stages: young foliage (young, white circles), maximum growth rate (mature, grey squares) and beginning of senescence (old, black diamonds). Each point represents the average value (n = 6) for a given plant species at a given ontogenetic stage. Regressions are based on average values and given across all species and ontogenetic stages in (a) (y = 0.19x-26.82, R^2^ = 0.23, p = 0.028) and for all ontogenetic stages (dashed line, y = -0.31x-19.26, R^2^ = 0.25, p = 0.021) as well as mature stage only in (b) (y = -0.21x-21.31, R^2^ = 0.83, p = 0.0045). Error bars indicate ±1SE.

Across all ontogenetic stages and species, δ^13^C_CO2-leaf_ ranged from about -30‰ to -22‰, showing larger variation than δ^13^C_CO2-soil_ (Table 2, S1 Table). ^13^C of leaf-respired CO_2_ was enriched compared to leaf biomass and phloem sap organic matter (Fig 1). However, as mentioned above, we only calculated Δ_R_ based on phloem sap organic matter (see below). Over all ontogenetic stages and species, δ^13^C_CO2-leaf_ (-26.0±0.3‰ overall mean±SE) was significantly lower (p<0.001) than δ^13^C_CO2-soil_ (-24.4±0.3‰ overall mean). δ^13^C_CO2-leaf_ was significantly affected by ontogenetic stage and species, but not by functional group (Table 3, S1 Fig). Furthermore, over all ontogenetic stages and species, δ^13^C_CO2-leaf_ was positively related with leaf biomass (R^2^ = 0.23, p = 0.028, Fig 2A) and negatively related with Δ_i_ (R^2^ = 0.25, p = 0.021). However, the latter relationship was driven only by mature stage plants (R^2^ = 0.83, p = 0.0045, Fig 2B).

**Table 2:** δ^13^C values of leaf-respired CO_2_ (δ^13^C_CO2-leaf_), soil-respired CO_2_ (δ^13^C_CO2-soil_), microbial biomass (δ^13^C_mic_) and bulk soil δ^13^C (δ^13^C_soil_) at three ontogenetic stages (young foliage, “young”; maximum growth rate, “mature”; beginning of senescence, “old”) in seven species (*Arrhenatherum elatius*, *Dactylis glomerata*, *Hordeum vulgare*, *Lolium perenne*, *Medicago sativa*, *Trifolium pratense*, *Triticum aestivum*). Values are given as mean±1SE (n = 6). Within one column, different letters indicate significant differences between ontogenetic stages within a species (p≤0.05). Note that only significant differences are shown.

Ontogenetic stage	Species	δ^13^C_CO2-leaf_	δ^13^C_CO2-soil_	δ^13^C_mic_	δ^13^C_soil_
Young					
	*Arrhenatherum*	-24.08±0.40	-24.27±0.66	-23.68±0.19	-25.39±0.09
	*Dactylis*	-27.19±1.13	-24.20±0.54	-24.26±0.59	-25.49±0.02
	*Hordeum*	-27.60±1.39	-22.31^a^±0.70	-21.89^a^±0.13	-25.35±0.08
	*Lolium*	-26.58±0.63	-24.40±0.52	-23.08±0.23	-25.37±0.12
	*Medicago*	-29.69±0.40	-26.83±1.22	-23.15±0.31	-25.38±0.07
	*Trifolium*	-25.69±0.40	-25.39±0.83	-25.63±0.56	-25.42±0.10
	*Triticum*	-26.10^b^±0.91	-21.36^a^±1.51	-21.31^a^±0.57	-25.29±0.09
Mature					
	*Arrhenatherum*	-25.84±0.99	-24.07±0.39	-23.47±1.31	-25.64±0.07
	*Dactylis*	-25.37±1.14	-25.09±0.74	-24.57±1.27	-25.43±0.05
	*Hordeum*	-26.31±0.96	-26.45^b^±0.44	-25.71^b^±0.56	-25.53±0.04
	*Lolium*	-26.25±0.82	-24.57±0.41	-25.10±0.33	-25.55±0.04
	*Medicago*	-26.35±1.04	-24.10±0.77	-25.41±0.46	-25.48±0.06
	*Trifolium*	-25.87±0.95	-23.38±0.90	-23.81±0.37	-25.49±0.10
	*Triticum*	-26.73^b^±0.64	-25.09^b^±0.67	-25.43^b^±1.26	-25.59±0.06
Old					
	*Arrhenatherum*	-25.55±0.77	-24.65±0.64	-24.60±0.46	-25.61±0.05
	*Dactylis*	-24.95±1.44	-23.34±0.42	-25.23±0.39	-25.47±0.08
	*Hordeum*	-22.35±2.26	-25.00^b^±0.60	-24.84^ab^±0.36	-25.42±0.13
	*Lolium*	-26.14±1.57	-25.05±0.84	-23.44±1.14	-25.51±0.06
	*Medicago*	-28.19±1.09	-24.96±0.14	-22.44±2.27	-25.57±0.03
	*Trifolium*	-25.30±0.93	-23.19±0.45	-25.33±0.72	-25.49±0.08
	*Triticum*	-23.69^a^±1.07	-24.80^ab^±0.71	-24.26^ab^±0.47	-25.49±0.05

**Table 3:** ANOVA results for δ^13^C values, and respiratory C isotope fractionation between phloem sap organic matter and leaf- and soil-respired CO_2_ (Δ_Rphloem-leaf_ and Δ_Rphloem-soil_, respectively). The variables are growth chamber, plant ontogenetic stage, functional group identity, and species identity (block, onto, group and species, respectively). F_x,y_, x refers to the degrees of freedom of the tested variable and y to the d.f. of the residuals.

Source of variation	δ^13^C_phloem_	δ^13^C_mic_	δ^13^C_CO2-leaf_	δ^13^C_CO2-soil_	Δ_Rphloem-leaf_	Δ_Rphloem-soil_
block	**F**_**5,78**_ = 2.49*	**F**_**5,79**_ = 2.40*	**F**_**5,86**_ = 2.91*	F_5,91_ = 1.06	**F**_**5,71**_ **= 2.20 .**	**F**_**5,76**_ **= 0.65 .**
onto	**F**_**2,78**_ = 19.87***	**F**_**2,79**_ = 7.99***	**F**_**2,86**_ = 4.11*	F_2,91_ = 0.66	**F**_**2,71**_ = 3.26*	**F**_**2,76**_ = 6.17**
group	**F**_**2,4**_ = 15.17*	F_2,4_ = 0.27	F_2,4_ = 0.88	F_2,4_ = 0.28	**F**_**2,4**_ = 9.63*	**F**_**2,4**_ = 9.56*
species	F_4,78_ = 1.08	F_4,79_ = 1.43	**F**_**4,86**_ = 3.56**	F_4,91_ = 1.60	F_4,71_ = 0.89	F_4,76_ = 0.86
onto:group	**F**_**4,8**_ = 14.6***	F_4,8_ = 2.25	F_4,8_ = 1.96	**F**_**4,8**_ = 18.90***	F_4,8_ = 0.83	**F**_**4,8**_ = 12.69**
onto:species	F_8,86_ = 0.26	**F**_**8,79**_ **= 1.97 .**	F_8,86_ = 1.53	F_8,91_ = 0.58	F_8,71_ = 1.66	F_8,76_ = 0.40

Significance levels

**.** 0.1≥*P*>0.05

* 0.05≥*P*>0.01

** 0.01≥*P*>0.001

*** 0.001≥*P*.

### Plant respiratory fractionation

**Fig 3:**
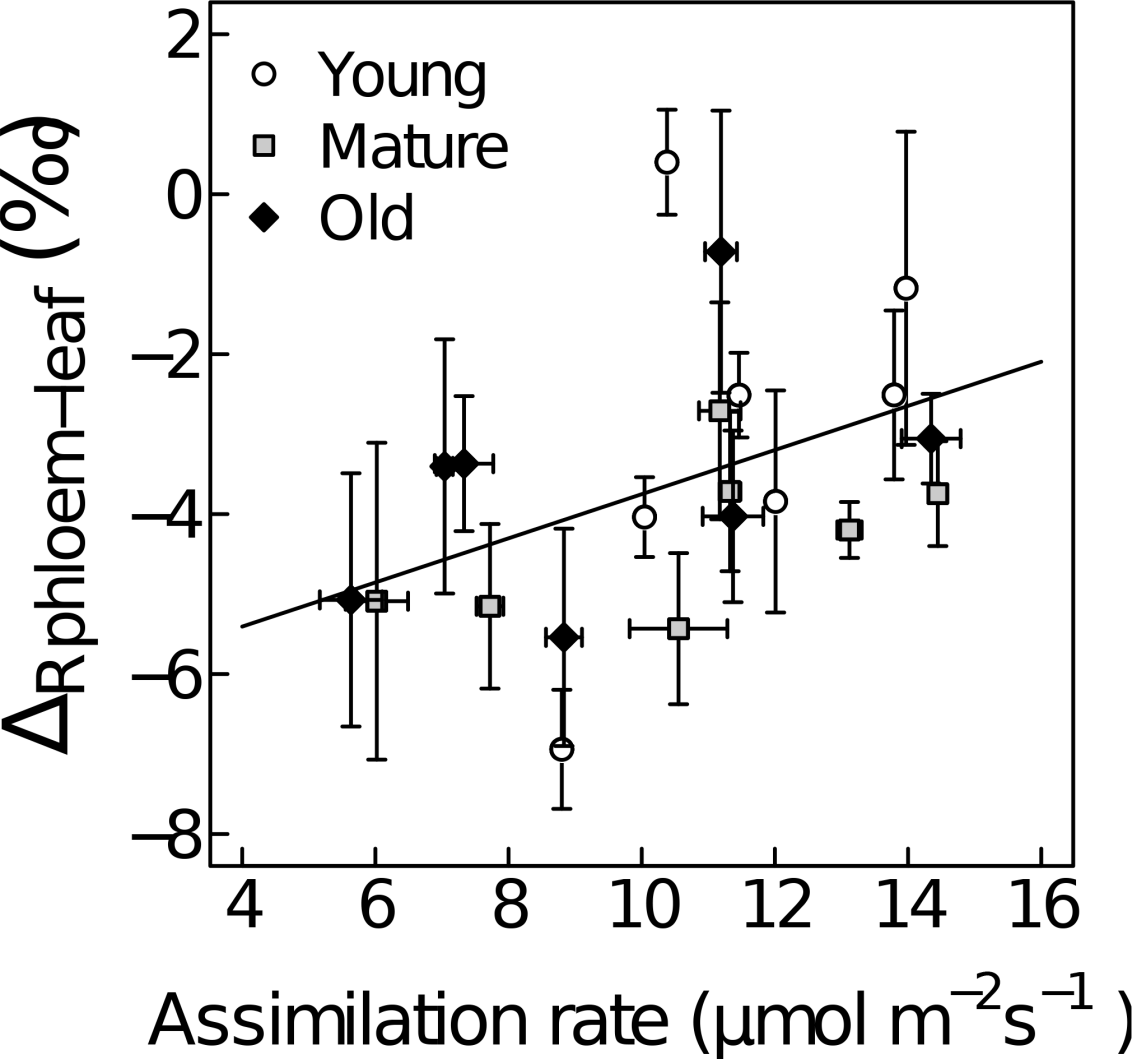
Response of respiratory C isotope fractionation (Δ_R_) between phloem sap organic matter and leaf-respired CO_2_ (Δ_Rphloem-leaf_) to leaf assimilation rate at three ontogenetic stages: young foliage (young, white circles), maximum growth rate (mature, grey squares) and beginning of senescence (old, black diamonds). Each point represents the average value (n = 6) for a given plant species at a given ontogenetic stage. The regression is given across all species and ontogenetic stages and based on the average values (y = 0.28x-6.51, R^2^ = 0.19, p = 0.051). Error bars indicate ±1SE.

Δ_Rphloem-leaf_ values were generally negative (Fig 3 and S2 Fig; except in young *Medicago*). Δ_Rphloem-leaf_ was significantly affected by ontogeny (Table 2, Table 4, overall mean±SE: -3.6±0.3‰), and differed significantly among functional groups (Table 3, S2 Fig). Δ_Rphloem-leaf_ tended to be positively related to assimilation rate (R^2^ = 0.19, p = 0.051; Fig 3).

**Table 4:** Respiratory C isotope fractionation (Δ_R_) at three ontogenetic stages (young foliage, “young”; maximum growth rate, “mature”; beginning of senescence, “old”) in seven species (*Arrhenatherum elatius*; *Dactylis glomerata*; *Hordeum vulgare*; *Lolium perenne*; *Medicago sativa*; *Trifolium pratense*; *Triticum aestivum*). Δ_R_ is calculated between phloem sap organic matter and leaf-respired CO_2_ (Δ_Rphloem-leaf_) as well as between phloem sap organic matter and soil-respired CO_2_ (Δ_Rphloem-soil_). Within one column, different letters indicate significant difference between ontogenetic stages within a species (p≤0.05). Note that only significant differences are shown.

Ontogenetic stage	Species	Δ_Rphloem-leaf_	Δ_Rphloem-soil_
Young			
	*Arrhenatherum*	-6.94^b^±0.74	-6.91±1.13
	*Dactylis*	-3.84±1.39	-6.96±0.90
	*Hordeum*	-1.18±1.96	-6.11±0.99
	*Lolium*	-4.04±0.50	-6.44±0.85
	*Medicago*	0.40^ab^±0.66	-1.87±1.38
	*Trifolium*	-2.51±0.53	-3.49±0.74
	*Triticum*	-2.51±1.06	-6.81±1.73
Mature			
	*Arrhenatherum*	-5.43^a^±0.94	-6.59±0.65
	*Dactylis*	-5.09±1.98	-6.43±0.96
	*Hordeum*	-4.20±0.35	-4.24±0.82
	*Lolium*	-5.15±1.03	-7.19±0.93
	*Medicago*	-3.72 ^b^±1.00	-6.05±1.10
	*Trifolium*	-2.71±1.36	-6.19±0.70
	*Triticum*	-3.74±0.66	-5.41±0.98
Old			
	*Arrhenatherum*	-3.37^a^±0.84	-4.30±1.26
	*Dactylis*	-5.54±1.36	-6.21±0.64
	*Hordeum*	-5.07±1.58	-2.44±0.78
	*Lolium*	-3.40±1.59	-4.55±0.86
	*Medicago*	-0.72^a^±1.76	-3.69±0.35
	*Trifolium*	-3.06±0.56	-5.28±0.55
	*Triticum*	-4.03±1.07	-2.87±0.94

Calculating plant respiratory fractionation based on other potential substrates, i.e., leaf biomass and photoassimilates, showed patterns that were generally similar to that of Δ_Rphloem-leaf_ for the seven studied species, despite a few exceptions particularly with photoassimilates as a source for respiration (S3 Table).

### Isotopic signature of microbial biomass and soil-respired CO_2_

δ^13^C values of microbial biomass ranged from about -25‰ to -21‰ (-23.9±0.2‰ overall mean, Fig 3), and were overall significantly more enriched (p<0.001) than bulk soil δ^13^C (-25.5±0.0‰ overall mean). δ^13^C_mic_ was significantly affected by ontogeny. No significant correlations were observed between any measured ecophysiological parameter and δ^13^C_mic_. Bulk soil δ^13^C was -25.48±0.01‰ (overall mean±se, Table 2) and was not affected by either ontogeny or the species growing in.

The ^13^C signal of soil CO_2_ efflux was enriched compared to all plant components and generally also compared to bulk soil, but not compared to microbial biomass (Fig 1). The effect of ontogeny on δ^13^C_CO2-soil_ differed among functional groups (Table 3, S1 Fig), driven by significant differences among ontogenetic stages that were found only in the crops *Triticum* and *Hordeum* (Table 2). No significant correlations were observed between measured leaf-gas exchange variables and δ^13^C_CO2-soil_.

### Belowground respiratory fractionation

Δ_Rphloem-soil_ values (overall mean±SE: -6.5±0.4‰,) were negative (Table 4, S2 and S3 Figs), indicating enrichment in ^13^C of respired CO_2_ compared to the putative respiration substrates. Δ_Rphloem-soil_ was significantly affected by ontogeny (Table 3, Table 4) and the interaction between functional group and ontogeny. Furthermore, Δ_Rphloem-soil_ tended to be negatively related to Δ_i_ (R^2^ = 0.19, p = 0.051) and positively to WUE (R^2^ = 0.16, p = 0.069). In contrast, Δ_Rphloem-soil_ was positively related to A/R_l_ for plants at the old stage (R^2^ = 0.68, p = 0.021).

Considering other potential substrates for belowground respiratory fractionation (i.e., leaf and root biomass, photoassimilates,) showed similar patterns of the ontogeny response as for Δ_Rphloem-soil_ for the seven species studied, despite a few exceptions, particularly when choosing photoassimilates as a source for respiration (S3 Table).

## Discussion

### Isotopic signature of plant-respired CO_2_

We found large, significant effects of ontogeny and species identity on δ^13^C_CO2-leaf_. The magnitude of change due to both ontogeny and species identity effects on the δ^13^C signal of respired CO_2_ was up to 7‰, and the associated respiratory fractionation reached up to 7‰ in Δ_Rphloem-leaf_. This is in agreement with the 6‰ increase in δ^13^C of leaf-respired CO_2_ during the first 22 days of growth of French beans and peanuts, documented in the–to our knowledge–only other studies about the impact of ontogeny on δ^13^C_CO2_ [3, 17, 18]. These values thus span a similar range as changes in the δ^13^C of leaf-respired CO_2_ resulting from changes in environmental conditions (e.g., about 8‰ in [40]). Thus, our results emphasise the relevance of accounting for ontogeny and species identity when using respiratory δ^13^C values to infer plant responses to changing environments. Furthermore, δ^13^C_CO2-leaf_ was more enriched than its potential substrate in all species and stages considered, indicating that respiratory fractionation in leaves should be accounted for when using δ^13^C to study respiration in C_3_ herbaceous species. The large ontogenetic and species effects that we found on δ^13^C_CO2-leaf_ were associated with significant changes in Δ_Rphloem-leaf_ driven by ontogeny and the identity of species or functional groups.

Short-term processes imprint the isotopic signature of photoassimilates that will be allocated to fast- or slow-turnover C pools. Long-term processes control the isotopic signature of slow-turnover C pools. Our results illustrate how both short- and long-term processes are involved in the changes in δ^13^C of respired CO_2_ with species and ontogenetic stages. In the short-term, δ^13^C_CO2-leaf_ was significantly negatively related to Δ_i_, especially at the mature stage, which is directly related to the isotopic signature of fast-turnover C pools [41–43]. The coupling between δ^13^C_CO2-leaf_ and Δ_i_ is in agreement with general predictions for annuals and fast-growing species with small C reserves [44], and suggests that a fraction of the newly-synthesized assimilates was likely directly respired by the leaf. Furthermore, Δ_i_ is mostly driven by A and g_s_: it is therefore highly dependent on plant regulation of gas exchange. Under controlled conditions, in the absence of day-to-day environmental variation, changes in leaf gas exchange rates are not driven by changes in climatic conditions, but are responding to changes in the ratio of plant C source to sink strengths [45–47], either directly, as decreasing C source:sink ratios will stimulate leaf gas exchange to meet the increasing C demand, or indirectly, as increasing C sinks will translate to increasing leaf and root biomass. Increasing leaf biomass in turn results in higher photosynthesis and transpiration, while increasing root biomass allows better access to water resource, which in turn can translate in higher transpiration, A and g_s_.

Therefore, the relationship between leaf gas exchange and Δ_i_ across species and ontogenetic stage suggests that species-related differences in δ^13^C_CO2-leaf_ could be related to shifts in the balance between C sources and sink strengths. Considering the possible fate of C once assimilated or released from storage, it can either be used to build biomass, be respired or exported. While information about the δ^13^C of exported C–particularly out of the plant (e.g. exudates)–are lacking, in the long-term, we found that δ^13^C_CO2-leaf_ became more enriched as the plants grew older. Even in the absence of the isotopic signature of exported C, this relationship between δ^13^C of C incorporated in biomass and respired is likely due to changes in the balance between maintenance and growth respiration as plant grew older. Indeed, young and growing plants synthetize large amount of proteins–in particular RuBisCO [48]–and cellulose, both of which generally slightly enriched in ^13^C compared to leaf organic matter [13], thus generating CO_2_ that is more ^13^C-depleted than bulk leaf material. At later growth stages, RuBisCO degradation [48] could result in the release of more enriched ^13^C-CO_2_.

### Plant respiratory fractionation and C balance

Over all species and ontogenetic stages, we found a positive relationship between Δ_Rphloem-leaf_ and A. Under our controlled experimental conditions, and within the limits set by the available light intensity and photosynthetic capacity (e.g., as a function of available RuBisCO), changes in the balance between C source and sink strengths were very likely influencing photosynthetic activity [45–47]. Our results suggest that increased C demand from respiring and growing tissues stimulate higher assimilation rates to meet the additional C costs and is associated with less fractionation. In a conifer forest, Ubierna & Marshall [14] also found that C allocation played a key role, but the relationship between A and the difference between δ^13^C_leaf_ and δ^13^C_CO2-leaf_ was negative. This relationship was attributed to changes in respiratory fractionation resulting from changes in available C that could be diverted to secondary metabolism. Although the relationship between secondary metabolism and δ^13^C respired CO_2_ has not been formally proven, this hypothesis is well supported by experimental data (see for example [4] for details). These opposite results might, however, be driven by the same underlying mechanism: higher C availability for secondary metabolism increases respiratory fractionation. Under field conditions, A is mostly regulated by environmental conditions and particularly light availability, while under constant day-to-day environmental conditions such as in our experiment, A will respond positively to an increase in C sink strength relative to C source. This can be both direct, i.e. increased carbohydrate demand by respiring and growing tissues stimulating C assimilation [45–47, 49], or indirect, when growing aboveground tissue leads to an increase in photosynthetically active tissue. Thus, under field conditions, in addition to covering respiratory C costs and growth, higher photosynthesis rates allow to have carbohydrates available for secondary metabolism, resulting in more apparent fractionation between δ^13^C of respired CO_2_ and δ^13^C of respiratory substrate [14]. However, when photosynthetic rate is stimulated by higher C sink strength, higher C demand for growth and maintenance respiration will lead to both higher A and less fractionation between respired CO_2_ and its substrate (smaller Δ_Rphloem-leaf_ in absolute value), since less C is available for secondary metabolism. Therefore, while our results only describe an ontogenetic trend among herbaceous C_3_ species and do not allow formal testing of the underlying mechanisms, they are consistent with the hypothesis that respiratory fractionation in leaves is likely responding to changes in the ratio between the strength of C sinks and sources.

### Isotopic signature of soil-respired CO_2_

At the timescale of our study, we found little overall effects of species, functional group or ontogeny on δ^13^C_CO2-soil_, in line with the expected low ontogenetic variability in δ^13^C of root-respired CO_2_ (see recent review by [17]). However, the ontogenetic pattern of δ^13^C_CO2-soil_ differed among functional groups (significant ontogeny × functional group interaction), as a significant ontogeny effect was detected in crops (namely in *Triticum* and *Hordeum*) but not in the other groups. δ^13^C_mic_ showed a response pattern that was similar to that of δ^13^C_CO2-soil_, suggesting that the crop-specific ontogenetic effects on the isotopic signature of soil CO_2_ emissions were, at least in part, shaped by the metabolic activity of the soil microbial community. Changes in microbial community structure have been associated with changes in the isotopic signature of CO_2_ respired by heterotrophs [50, 51]. Thus, our results suggest that plant-microbial interaction dynamics over time may differ between crops and the other functional groups included here.

The large soil organic matter pool most likely contributed as a substrate for microbial respiration and thus for soil CO_2_ efflux in our experiment, especially for plants in the young stage. Based on the relative plant assimilation and soil respiration rates at different ontogenetic stages, we can at least determine stages at which soil organic C may have been contributing to soil CO_2_ efflux. At the young stage, overall photosynthesis was likely not sufficient to sustain the total soil CO_2_ efflux, except in crops (S4 Table). At the mature stage, soil CO_2_ efflux could have been fuelled by assimilation in all species, except *Dactylis*. Finally, at the old stage, the overall assimilation flux was potentially sufficient to meet respiration costs, except for legumes. Thus, the discrimination associated with soil microbial respiratory processes may change with ontogeny, as a consequence of several processes, including shifts in the relative activity of different microbial populations [50, 51] or in the organic substrate preferentially metabolized [52], but our experimental setup does not allow to quantify these processes.

Additionally, the changes in δ^13^C_CO2-soil_ and associated changes in Δ_Rsoil-phloem_ with ontogeny and functional group were possibly driven by 1) changes in the relative contribution of plants to the overall soil CO_2_ efflux, both through root respiration and microbial respiration fed by plant rhizodeposits, and 2) changes in the fractionation between respired CO_2_ and respiratory substrate provided by the plants, i.e., phloem sap organic matter, either directly as a source for root respiration or indirectly as C exudates. Little information is available to discuss (1), especially in terms of root exudation. Nonetheless, the contribution of root-respired CO_2_ (and its isotopic signature) to total soil CO_2_ efflux and its signature is expected to increase with the development of the plant root system. The lack of a significant relationship in our study between Δ_Rsoil-phloem_ and aboveground biomass (the latter should be correlated with root biomass [53]) does not support an increased contribution of roots to total CO_2_ efflux with growth. However, our results support (2) as a mechanism shaping part of the ontogeny and functional group effects on δ^13^C_CO2-soil._ As expected, ontogeny strongly affected Δ_Rsoil-phloem_, since it also affected its putative substrate, δ^13^C_phloem_. Nevertheless, considering a different proxy for respiration substrate, such as root biomass, showed very similar results (S3 Table). Thus, the Δ_Rsoil-phloem_ response to ontogeny was not solely driven by change in δ^13^C_phloem_, but reflected actual changes in respiratory processes. In contrast, we detected no overall ontogeny effects on δ^13^C_CO2-soil_, suggesting that although ontogeny affects the ^13^C signature of plant material, these changes do not propagate to the isotopic signature of soil respiration.

Moreover, our study showed consistent relationships on different time scales between Δ_Rsoil-phloem_ and leaf gas exchange. Δ_Rsoil-phloem_ was negatively related to Δ_i_ (i.e., dependent on A and g_s_) and positively related to the ratio of assimilation to leaf respiration (A/R_l_) at the old stage. Note that similar relationships were found when considering another proxy than phloem as respiration substrate, indicating that these results were not an artefact due to the correlation between δ^13^C of phloem and leaf gas exchange. Both relations suggest that, in agreement with results for aboveground tissues, increased C assimilation (higher A, resulting in both higher A/R_l_ and lower Δ_i_) leads to smaller respiratory fractionation in absolute values, most likely since most assimilated C is directed towards primary rather than secondary metabolism. This hypothesis is supported by several studies that documented the importance of aboveground controls of C allocation to root respiration ([54] and references therein). Additionally, C fixation by PEPc in belowground plant tissues might also play an important role in the isotopic signature of respiratory substrates in roots [17] and thus lead to an apparent fractionation between phloem sap organic matter and respired CO_2_.

The ontogeny effect on Δ_Rsoil-phloem_ was specific to each functional group. In particular, crops showed a significant decrease in absolute values between younger and older stages, while little differences were found in forage grasses and legumes. Crops, unlike legumes and forage grasses, are selected for grain rather than vegetative biomass production. Despite grain filling did not take place in this study (see above), C allocation and nutrient uptake in crops may have been switching towards storage (for example as sucrose and fructans in the stem [55] and references therein) in preparation for grain filling, possibly leading to the measured changes in apparent respiratory C fractionation and consequently in δ^13^C_CO2-soil_, efflux. Furthermore, crop is the only functional group for which total assimilation was sufficient to cover total C lost through respiration at every ontogenetic stage (S4 Table). This is consistent with a tighter coupling between respiration fractionation in soil and plant C dynamics in crops than in other functional groups.

### Conclusion

Our results show that biological factors (i.e., ontogeny and species identity) can strongly affect the carbon isotopic signature of respired CO_2_ and respiratory fractionation. These factors need to be accounted for in studies inferring physiological responses of plants to their biotic and abiotic environment based on δ^13^C of respired CO_2_ and respiratory fractionation. Furthermore, we show that these ontogeny-related changes in δ^13^C of respired CO_2_ and respiratory fractionation are associated with changes in physiological processes involved in the plant C budget and particularly with changes in photosynthesis, biomass and respiration. Therefore, our results suggest that ontogeny-related changes in the ratio of plant internal C source to sink strengths drove the measured changes in δ^13^C of respired CO_2_ and respiratory fractionation.

## Supporting Information

S1 Fig δ^13^C values of leaf-respired CO_2_ and soil-respired CO_2_ at three ontogenetic stages in three functional groups. (DOCX)

S2 Fig Respiratory carbon isotope fractionation (Δ_R_) in three functional groups. (DOCX)

S3 Fig Response of Δ_Rphloem-soil_ to Δ_i_ and to A/R_l_. (DOCX)

S1 Table δ^13^C values (in per mil) of leaf biomass, root biomass, phloem organic matter as well as leaf and soil respired CO_2_ for different plant species at three ontogenetic stages. (DOCX)

S2 Table ANOVA results for R_l_, A, g_s_, Δ_i_, B, LA and R_s_. (DOCX)

S3 Table Respiratory C isotope fractionation (Δ_R_) at three ontogenetic stages in seven species. (DOCX)

S4 Table Estimated average daily C balance of pots. (DOCX)

## Abbreviation Name

a: Fractionation occurring during CO_2_ diffusion in air through the stomatal pore (4.4‰)
A: Assimilation rate [μmol m^-2^ s^-1^]
b: Net fractionation caused by carboxylation (27‰ was used here, see text for details)
B: Total dry aboveground biomass [g]
c_a_: Ambient CO_2_ concentration [μmol CO_2_ mol air^-1^]
c_i_: Substomatal CO_2_ concentration [μmol CO_2_ mol air^-1^]
g_s_: Stomatal conductance to H2O [mmol H2O m^-2^ s^-1^]
LA: Total leaf surface area [cm^2^]
PAR: Photosynthetically active radiation [μmol m^-2^ s^-1^]
R_l_: Leaf dark respiration rate [μmol m^-2^ s^-1^]
R_s_: Soil respiration rate [μmol m^-2^ s^-1^]
Δ: Isotope discrimination
Δ_i_: Predicted photosynthetic C isotope discrimination assuming infinite mesophyll conductance and neglect the effect of (photo)respiration [‰]
Δ_p_: Observed C isotope discrimination between atmospheric CO_2_ and the considered plant part (leaf biomass, bulk phloem sap organic matter, root biomass) [‰]
Δ_R_: Respiratory C isotope fractionation [‰]
δ^13^C_CO2-leaf_: Isotope composition of leaf-respired CO_2_ [‰]
δ^13^C_CO2-soil_: Isotope composition of soil CO_2_ efflux [‰]
δ^13^C_leaf_: δ^13^C of leaf total organic matter [‰]
δ^13^C_mic_: Isotope composition of soil microbial biomass C [‰]
δ^13^C_phloem_: δ^13^C of bulk phloem sap organic matter [‰]
δ^13^C_photo_: δ^13^C of new photoassimilates [‰]
δ^13^C_root_: δ^13^C of root total organic matter [‰]
Δ_Rphloem-leaf_: Respiratory C isotope fractionation between bulk phloem sap organic matter and leaf-respired CO_2_ [‰]
Δ_Rphloem-soil_: Respiratory C isotope fractionation between bulk phloem sap organic matter and soil CO_2_ efflux [‰]
