# Supplementary material for: Effects of Ontogeny on δ13C of Plant- and Soil-Respired CO2 and on Respiratory Carbon Fractionation in C3 Herbaceous Species
Source: PLoS One. 2016 Mar 24;11(3):e0151583. doi: 10.1371/journal.pone.0151583 (PMC4807002; doi:10.1371/journal.pone.0151583)
Supplement: S1 Fig — (DOCX) [file pone.0151583.s001.docx]

**Figure S1:** δ^13^C values of leaf-respired CO_2_ (left column:) and soil-respired CO_2_ (right column: at three ontogenetic stages (young foliage, “young”, white bars; maximum growth rate, “mature”, grey bars; beginning of senescence, “old”, black bars) in three functional groups (forage grasses, “grasses”, A and B,; “legumes” C and D; “crops” E and F). Bars indicate mean±1SE (n=6). Within a panel, bars sharing the same letter are not significantly different from each other (p≥0.05, Tukey HSD test). Note that only significant differences are shown.
