# Supplementary material for: Effects of Ontogeny on δ13C of Plant- and Soil-Respired CO2 and on Respiratory Carbon Fractionation in C3 Herbaceous Species
Source: PLoS One. 2016 Mar 24;11(3):e0151583. doi: 10.1371/journal.pone.0151583 (PMC4807002; doi:10.1371/journal.pone.0151583)
Supplement: S2 Fig — (DOCX) [file pone.0151583.s002.docx]

**Figure S2:** Respiratory carbon isotope fractionation (Δ_R_) in three functional groups (forage grasses, “grasses”, A and B; “legumes”, C and D; “crops”, E and F). Δ_R_ is calculated between the following sources and products: phloem organic matter and leaf-respired CO_2_ (Δ_Rphloem-leaf_, columns A, C and E), phloem organic matter and soil-respired CO_2_ (Δ_Rphloem-soil_, columns B, D and F at three ontogenetic stages (young foliage, “young”, white bars; maximum growth rate, “mature”, grey bars; beginning of senescence, “old”, black bars). Bars indicate mean±1SE (n=6). Within a panel, bars sharing the same letter are not significantly different from each other (p≥0.05, Tukey HSD test). Note that only significant differences are shown.
