# Supplementary material for: Effects of Ontogeny on δ13C of Plant- and Soil-Respired CO2 and on Respiratory Carbon Fractionation in C3 Herbaceous Species
Source: PLoS One. 2016 Mar 24;11(3):e0151583. doi: 10.1371/journal.pone.0151583 (PMC4807002; doi:10.1371/journal.pone.0151583)
Supplement: S3 Fig — (DOCX) [file pone.0151583.s003.docx]

**Figure S3**: Response of Δ_Rphloem-soil_ to Δ_i_ (a) at three ontogenetic stages: young foliage (young, white circles), maximum growth rate (mature, gray squares) and beginning of senescence (old, black diamonds), and to A/R_l_ (b) at the very beginning of senescence (old, black diamonds). Each point represents the average value (n=6) for a given plant species at a given ontogenetic stage. Regressions are based on the average values and given across all species and ontogenetic stages in (a) (y=-0.27+0.82, R^2^=0.19, p=0.051) but for mature stage only in (b) (y=0.051x-5.51, R^2^=0.68, p=0.022). Error bars indicate ±1SE.
