# Supplementary material for: Effects of Ontogeny on δ13C of Plant- and Soil-Respired CO2 and on Respiratory Carbon Fractionation in C3 Herbaceous Species
Source: PLoS One. 2016 Mar 24;11(3):e0151583. doi: 10.1371/journal.pone.0151583 (PMC4807002; doi:10.1371/journal.pone.0151583)
Supplement: S1 Table — (DOCX) [file pone.0151583.s004.docx]

**Table S1:** δ^13^C values (in per mil) of leaf biomass, root biomass, phloem organic matter as well as leaf and soil respired CO_2_ for different plant species at three ontogenetic stages (young foliage stage, maximum growth rate stage and beginning of senescence stage, indicated by young, mature and old, respectively). Values are mean±1SE (n=6) and are corrected for changes in δ^13^C values of background CO_2­_ averaged over the corresponding growth period (δ^13^C­_air_). δ^13^C_phloem_, δ^13^C_leaf,_ δ^13^C_root_ and δ^13^C­_air_ were originally published in Salmon *et al.* (2011).

| Stage | Species | δ^13^C_leaf_ | | | δ^13^C_root_ | | | δ^13^C_phloem_ | | | δ^13^C_CO2-leaf_ | | | δ^13^C_CO2-soil_ | | |  | δ^13^C­_air_ |
| --- | --- | --- | --- | --- | --- | --- | --- | --- | --- | --- | --- | --- | --- | --- | --- | --- | --- | --- |
| Young |  |  |  |  |  |  |  |  |  |  |  |  |  |  |  |  |  |  |
|  | *Arrhenatherum* | -31.9 | ± | 0.5 | -30.3 | ± | 0.5 | -31.7 | ± | 0.5 | -24.1 | ± | 0.4 | -24.3 | ± | 0.7 |  | -9.3 |
|  | *Dactylis* | -31.9 | ± | 0.3 | -30.6 | ± | 0.4 | -31.8 | ± | 0.5 | -27.2 | ± | 1.1 | -24.2 | ± | 0.5 |  | -8.5 |
|  | *Hordeum* | -29.7 | ± | 0.2 | -26.3 | ± | 0.1 | -29.2 | ± | 0.5 | -27.6 | ± | 1.4 | -22.3 | ± | 0.7 |  | -9.4 |
|  | *Lolium* | -33.3 | ± | 0.5 | -30.9 | ± | 0.4 | -31.6 | ± | 0.5 | -26.6 | ± | 0.6 | -24.4 | ± | 0.5 |  | -9.3 |
|  | *Medicago* | -33.1 | ± | 0.4 | -31.0 | ± | 0.3 | -30.4 | ± | 0.6 | -29.7 | ± | 0.4 | -26.8 | ± | 1.2 |  | -9.3 |
|  | *Trifolium* | -31.1 | ± | 0.2 | -29.8 | ± | 0.3 | -28.9 | ± | 0.0 | -25.7 | ± | 0.4 | -25.4 | ± | 0.8 |  | -8.5 |
|  | *Triticum* | -29.5 | ± | 0.2 | -26.6 | ± | 0.1 | -29.5 | ± | 0.2 | -26.1 | ± | 0.9 | -21.4 | ± | 1.5 |  | -9.4 |
| Mature |  |  |  |  |  |  |  |  |  |  |  |  |  |  |  |  |  |  |
|  | *Arrhenatherum* | -31.3 | ± | 0.6 | -29.7 | ± | 0.7 | -31.4 | ± | 0.3 | -25.8 | ± | 1.0 | -24.1 | ± | 0.4 |  | -8.3 |
|  | *Dactylis* | -31.9 | ± | 0.2 | -29.7 | ± | 0.2 | -31.6 | ± | 0.8 | -25.4 | ± | 1.1 | -25.1 | ± | 0.7 |  | -9.2 |
|  | *Hordeum* | -32.0 | ± | 0.4 | -30.9 | ± | 0.4 | -31.8 | ± | 0.8 | -26.3 | ± | 1.0 | -26.4 | ± | 0.4 |  | -8.4 |
|  | *Lolium* | -32.5 | ± | 0.7 | -31.2 | ± | 0.7 | -32.3 | ± | 0.5 | -26.3 | ± | 0.8 | -24.6 | ± | 0.4 |  | -8.4 |
|  | *Medicago* | -32.6 | ± | 0.3 | -31.4 | ± | 0.3 | -30.4 | ± | 0.4 | -26.3 | ± | 1.0 | -24.1 | ± | 0.8 |  | -8.4 |
|  | *Trifolium* | -31.3 | ± | 0.2 | -29.4 | ± | 0.2 | -29.5 | ± | 0.3 | -25.9 | ± | 0.9 | -23.4 | ± | 0.9 |  | -9.2 |
|  | *Triticum* | -31.7 | ± | 0.3 | -30.7 | ± | 0.3 | -31.5 | ± | 0.6 | -26.7 | ± | 0.6 | -25.1 | ± | 0.7 |  | -8.5 |
| Old |  |  |  |  |  |  |  |  |  |  |  |  |  |  |  |  |  |  |
|  | *Arrhenatherum* | -29.8 | ± | 0.7 | -28.5 | ± | 0.8 | -29.6 | ± | 1.1 | -25.6 | ± | 0.8 | -24.7 | ± | 0.6 |  | -7.7 |
|  | *Dactylis* | -29.9 | ± | 0.7 | -28.7 | ± | 0.7 | -30.1 | ± | 0.8 | -24.9 | ± | 1.4 | -23.3 | ± | 0.4 |  | -7.7 |
|  | *Hordeum* | -29.1 | ± | 0.6 | -27.7 | ± | 0.5 | -28.1 | ± | 0.5 | -22.3 | ± | 2.3 | -25.0 | ± | 0.6 |  | -7.7 |
|  | *Lolium* | -30.8 | ± | 0.6 | -29.8 | ± | 0.7 | -30.2 | ± | 0.7 | -26.1 | ± | 1.6 | -25.0 | ± | 0.8 |  | -7.7 |
|  | *Medicago* | -31.3 | ± | 0.7 | -29.9 | ± | 0.8 | -29.5 | ± | 0.4 | -28.2 | ± | 1.1 | -25.0 | ± | 0.1 |  | -7.7 |
|  | *Trifolium* | -30.8 | ± | 0.8 | -29.3 | ± | 0.8 | -29.0 | ± | 0.7 | -25.3 | ± | 0.9 | -23.2 | ± | 0.5 |  | -7.6 |
|  | *Triticum* | -27.3 | ± | 0.4 | -25.4 | ± | 0.3 | -28.3 | ± | 0.8 | -23.7 | ± | 1.1 | -24.8 | ± | 0.7 |  | -7.7 |
