# Supplementary material for: Effects of Ontogeny on δ13C of Plant- and Soil-Respired CO2 and on Respiratory Carbon Fractionation in C3 Herbaceous Species
Source: PLoS One. 2016 Mar 24;11(3):e0151583. doi: 10.1371/journal.pone.0151583 (PMC4807002; doi:10.1371/journal.pone.0151583)
Supplement: S2 Table — (DOCX) [file pone.0151583.s005.docx]

**Table S2:** ANOVA results for R_l_, A, g_s_, Δ_i_, B, LA and R_s_. The variables are growth chamber, plant ontogenetic stage, functional group identity and species identity (block, onto, group and species, respectively). F_x,y_, x refers to the degrees of freedom of the tested variable and y to the d.f. of the residuals. Significance levels: . 0.1≥*P*>0.05, 0.05≥*P*>0.01, ** 0.01≥*P*>0.001, *** 0.001≥*P*.

| Source of variation | R_l_ | A | g_s_ | Δ_i_ | B | LA | R_s_ |
| --- | --- | --- | --- | --- | --- | --- | --- |
| block | F_2,38_=0.1 | F_2,38_=0.4 | F_2,38_=0.5 | F_2,38_=0.7 | F_2,40_=0.4 | F_2,40_=0.9 | F_2,40_=0.3 |
| onto | F_2,38_=2.7 | **F_2,38_=5.3**** | **F_2,38_=32.9***** | **F_2,38_=19.6***** | **F_2,40_=272.3***** | **F_2,40_=95.2***** | **F_2,40_=12.1***** |
| group | **F_2,4_=10.2*** | **F_2,4_=8.7*** | **F_2,4_=7.7*** | F_2,4_=0.0 | **F_2,4_=28.2**** | F_2,4_=2.6 | **F_2,4_=7.7*** |
| species | F_4,38_=1.5 | **F_4,38_=2.3.** | **F_4,38_=5.1**** | **F_4,38_=2.4.** | F_4,40_=1.5 | **F_4,40_=6.8***** | F_4,40_=1.1 |
| onto:group | F_4,8_=2.1 | F_4,8_=2.6 | **F_4,8_=6.2*** | **F_4,8_=3.1.** | **F_4,8_=3.8*** | F_4,8_=1.8 | **F_4,8_=6.0*** |
| onto:species | **F_8,38_=5.4***** | **F_8,38_=2.6*** | **F_8,38_=3.5**** | **F_8,38_=3.7**** | **F_8,40_=3.8**** | **F_8,40_=3.7**** | F_8,40_=0.6 |
