# Supplementary material for: Effects of Ontogeny on δ13C of Plant- and Soil-Respired CO2 and on Respiratory Carbon Fractionation in C3 Herbaceous Species
Source: PLoS One. 2016 Mar 24;11(3):e0151583. doi: 10.1371/journal.pone.0151583 (PMC4807002; doi:10.1371/journal.pone.0151583)
Supplement: S3 Table — (DOCX) [file pone.0151583.s006.docx]

**Table S3:** Respiratory C isotope fractionation (Δ_R_) at three ontogenetic stages (young foliage, “young”; maximum growth rate, “mature”; beginning of senescence, “old”) in seven species (*Arrhenatherum elatius*; *Dactylis glomerata*; *Hordeum vulgare*; *Lolium perenne*; *Medicago sativa*; *Trifolium pratense*; *Triticum aestivum*). Δ_R_ is calculated between the following sources and products: between leaf biomass and leaf-respired CO_2_ (Δ_Rleaf-leaf_), phloem organic matter and leaf-respired CO_2_ (Δ_Rphloem-leaf_), new photoassimilates and leaf-respired CO_2_ (Δ_Rphoto-leaf_), leaf biomass and soil-respired CO_2_ (Δ_Rleaf-soil_), phloem organic matter and soil-respired CO_2_ (Δ_Rphloem-soil_), new photoassimilates and soil-respired CO_2_ (Δ_Rphoto-soil_) and root biomass and soil-respired CO_2_ (Δ_Rroot-soil_). The isotopic signature of new photoassimilates (δ^13^C_photo_) was calculated using equations 3 and 5. Within one column, different letters indicate significant differences between ontogenetic stages within a species (p≤0.05). Note that only significant differences are shown.

| Stage | Species | Δ_Rleaf-leaf_ | | |  | Δ_Rphloem-leaf_ | | |  | Δ_Rphoto-leaf_ | | |  | Δ_Rleaf-soil_ | | |  | Δ_Rphloem-soil_ | | |  | Δ_Rphoto-soil_ | | |  | Δ_Rroot-soil_ | | |  |
| --- | --- | --- | --- | --- | --- | --- | --- | --- | --- | --- | --- | --- | --- | --- | --- | --- | --- | --- | --- | --- | --- | --- | --- | --- | --- | --- | --- | --- | --- |
| Young |  |  |  |  |  |  |  |  |  |  |  |  |  |  |  |  |  |  |  |  |  |  |  |  |  |  |  |  |  |
|  | *Arrhenatherum* | -7.13^b^ | ± | 0.68 |  | -6.94^b^ | ± | 0.74 |  | -8.1 | ± | 0.40 |  | -7.76 | ± | 0.93 |  | -6.91 | ± | 1.13 |  | -7.89 | ± | 0.66 |  | -5.37 | ± | 0.81 |  |
|  | *Dactylis* | -3.88 | ± | 1.16 |  | -3.84 | ± | 1.39 |  | -4.1 | ± | 1.15 |  | -7.77 | ± | 0.75 |  | -6.96 | ± | 0.90 |  | -7.14^b^ | ± | 0.55 |  | -5.75 | ± | 0.80 |  |
|  | *Hordeum* | -1.13 | ± | 1.48 |  | -1.18 | ± | 1.96 |  | -4.3 | ± | 1.40 |  | -7.48^b^ | ± | 0.61 |  | -6.11 | ± | 0.99 |  | -9.67^c^ | ± | 0.70 |  | -3.38 | ± | 0.71 |  |
|  | *Lolium* | -5.88 | ± | 0.59 |  | -4.04 | ± | 0.50 |  | -5.3 | ± | 0.64 |  | -9.02^b^ | ± | 0.78 |  | -6.44 | ± | 0.85 |  | -7.46-^b^ | ± | 0.52 |  | -5.82 | ± | 0.66 |  |
|  | *Medicago* | -2.40 | ± | 0.43 |  | 0.40^ab^ | ± | 0.66 |  | -1.9^a^ | ± | 0.40 |  | -6.35 | ± | 1.14 |  | -1.87 | ± | 1.38 |  | -4.81 | ± | 1.23 |  | -3.36 | ± | 1.12 |  |
|  | *Trifolium* | -4.72 | ± | 0.24 |  | -2.51 | ± | 0.53 |  | -3.0 | ± | 0.40 |  | -5.83 | ± | 0.75 |  | -3.49 | ± | 0.74 |  | -3.32^a^ | ± | 0.85 |  | -3.72 | ± | 0.74 |  |
|  | *Triticum* | -2.60 | ± | 1.10 |  | -2.51 | ± | 1.06 |  | -6.6 | ± | 0.92 |  | -8.29^b^ | ± | 1.61 |  | -6.81 | ± | 1.73 |  | -11.39^b^ | ± | 1.51 |  | -4.68^b^ | ± | 1.57 |  |
| Mature |  |  |  |  |  |  |  |  |  |  |  |  |  |  |  |  |  |  |  |  |  |  |  |  |  |  |  |  |  |
|  | *Arrhenatherum* | -4.76^ab^ | ± | 0.86 |  | -5.43^a^ | ± | 0.94 |  | -2.5 | ± | 1.01 |  | -7.36 | ± | 0.83 |  | -6.59 | ± | 0.65 |  | -4.26 | ± | 0.39 |  | -4.97 | ± | 0.95 |  |
|  | *Dactylis* | -5.74 | ± | 1.25 |  | -5.09 | ± | 1.98 |  | -2.1 | ± | 1.16 |  | -6.89 | ± | 0.78 |  | -6.43 | ± | 0.96 |  | -2.37^a^ | ± | 0.75 |  | -3.94 | ± | 0.80 |  |
|  | *Hordeum* | -4.94 | ± | 0.73 |  | -4.20 | ± | 0.35 |  | -5.8 | ± | 0.97 |  | -5.65^b^ | ± | 0.56 |  | -4.24 | ± | 0.82 |  | -5.66^b^ | ± | 0.45 |  | -3.73 | ± | 0.62 |  |
|  | *Lolium* | -5.50 | ± | 0.82 |  | -5.15 | ± | 1.03 |  | -3.8 | ± | 0.83 |  | -8.05^b^ | ± | 1.02 |  | -7.19 | ± | 0.93 |  | -5.52^b^ | ± | 0.41 |  | -5.94 | ± | 1.04 |  |
|  | *Medicago* | -5.48 | ± | 1.11 |  | -3.72 ^b^ | ± | 1.00 |  | -3.8^b^ | ± | 1.05 |  | -8.61 | ± | 0.71 |  | -6.05 | ± | 1.10 |  | -6.09 | ± | 0.78 |  | -6.68 | ± | 0.76 |  |
|  | *Trifolium* | -4.77 | ± | 0.88 |  | -2.71 | ± | 1.36 |  | -3.8 | ± | 0.96 |  | -8.07 | ± | 0.83 |  | -6.19 | ± | 0.70 |  | -6.35^b^ | ± | 0.91 |  | -5.37 | ± | 0.85 |  |
|  | *Triticum* | -4.23 | ± | 0.55 |  | -3.74 | ± | 0.66 |  | -5.6 | ± | 0.65 |  | -6.74^b^ | ± | 0.75 |  | -5.41 | ± | 0.98 |  | -7.21 | ± | 0.67 |  | -4.90^b^ | ± | 0.76 |  |
| Old |  |  |  |  |  |  |  |  |  |  |  |  |  |  |  |  |  |  |  |  |  |  |  |  |  |  |  |  |  |
|  | *Arrhenatherum* | -3.64^a^ | ± | 0.59 |  | -3.37^a^ | ± | 0.84 |  | -5.2 | ± | 0.78 |  | -5.28 | ± | 1.05 |  | -4.30 | ± | 1.26 |  | -6.09 | ± | 0.64 |  | -3.20 | ± | 1.18 |  |
|  | *Dactylis* | -4.47 | ± | 0.67 |  | -5.54 | ± | 1.36 |  | -4.9 | ± | 1.45 |  | -6.70 | ± | 0.60 |  | -6.21 | ± | 0.64 |  | -6.54^b^ | ± | 0.43 |  | -4.79 | ± | 0.55 |  |
|  | *Hordeum* | -6.57 | ± | 1.33 |  | -5.07 | ± | 1.58 |  | 0.1 | ± | 2.30 |  | -4.16^a^ | ± | 0.74 |  | -2.44 | ± | 0.78 |  | 2.80^a^ | ± | 0.62 |  | -2.08 | ± | 0.78 |  |
|  | *Lolium* | -3.95 | ± | 1.14 |  | -3.40 | ± | 1.59 |  | -1.4 | ± | 1.60 |  | -5.83^a^ | ± | 0.72 |  | -4.55 | ± | 0.86 |  | -2.56^a^ | ± | 0.86 |  | -4.15 | ± | 0.91 |  |
|  | *Medicago* | -2.27 | ± | 0.55 |  | -0.72^a^ | ± | 1.76 |  | 0.6^ab^ | ± | 1.12 |  | -6.40 | ± | 0.70 |  | -3.69 | ± | 0.35 |  | -2.65 | ± | 0.14 |  | -4.33 | ± | 0.76 |  |
|  | *Trifolium* | -4.96 | ± | 0.38 |  | -3.06 | ± | 0.56 |  | -6.1 | ± | 0.94 |  | -7.71 | ± | 0.74 |  | -5.28 | ± | 0.55 |  | -8.24^b^ | ± | 0.46 |  | -5.58 | ± | 0.76 |  |
|  | *Triticum* | -3.07 | ± | 0.71 |  | -4.03 | ± | 1.07 |  | -4.4 | ± | 1.08 |  | -2.56^a^ | ± | 0.37 |  | -2.87 | ± | 0.94 |  | -3.31 | ± | 0.72 |  | 0.03^a^ | ± | 0.49 |  |
