# Supplementary material for: Effects of Ontogeny on δ13C of Plant- and Soil-Respired CO2 and on Respiratory Carbon Fractionation in C3 Herbaceous Species
Source: PLoS One. 2016 Mar 24;11(3):e0151583. doi: 10.1371/journal.pone.0151583 (PMC4807002; doi:10.1371/journal.pone.0151583)
Supplement: S4 Table — (DOCX) [file pone.0151583.s007.docx]

**Table S4:** Estimated average daily C balance of pots (i.e. 16 plants and soils). C balance is equal to total photosynthesis minus total respiratory C losses:

C balance= A*LA*light.secs + A/2*LA*dusk.dawn.secs - (R_l_*LA*dark.secs + R_s_*pot.area*day.secs)

Data are the average values presented in Table 1. pot.area=0.0342m^2^. light.secs is the duration of the light period (14 h = 50400 seconds). Dusk.dawn.secs is the duration of the dusk and dawn periods (3600s each, thus 7200s), dark.secs is the duration of the dark period (8 h = 28800), day.secs is the number of seconds per 24h (i.e. 86400 seconds).

Note that this simple calculation likely overestimates average daily C balance, as it neglects stem respiration (but also stem assimilation), decreased photosynthetic activity of senescent leaves and assumes that all leaf area is equally exposed to maximum light.

| Ontogenetic stage | Species | C balance  (mmol day^-1^) |
| --- | --- | --- |
| Young |  |  |
|  | *Arrhenatherum* | -4.8 |
|  | *Dactylis* | -4.6 |
|  | *Hordeum* | 9.2 |
|  | *Lolium* | -7.6 |
|  | *Medicago* | -4.0 |
|  | *Trifolium* | -2.7 |
|  | *Triticum* | 8.5 |
| Mature |  |  |
|  | *Arrhenatherum* | 90.3 |
|  | *Dactylis* | -17.8 |
|  | *Hordeum* | 88.4 |
|  | *Lolium* | 47.8 |
|  | *Medicago* | 26.0 |
|  | *Trifolium* | 14.2 |
|  | *Triticum* | 66.7 |
| Old |  |  |
|  | *Arrhenatherum* | 76.9 |
|  | *Dactylis* | 73.3 |
|  | *Hordeum* | 49.8 |
|  | *Lolium* | 19.0 |
|  | *Medicago* | -18.6 |
|  | *Trifolium* | -54.2 |
|  | *Triticum* | 141.0 |
